# Eclectic Department

**Published:** 1856-11

**Authors:** 


London, Sept. 12th, 1856.

To the Editor of the American Medical Monthly.

Dear Sir: — After a pleasant trip in the *Baltic*, I find myself again in London, with one day to spare before continuing my journey to Vienna, where I expect to be by the 17th instant.

I spent this morning with my friend, Benjamin W. Richardson, whose researches, proving ammonia to be the constituent of the blood, which kept it in the fluid state, has won for him the last Astley Cooper Prize. The Doctor showed me some very interesting and really surprising experiments, clearly proving the existence of ammonia in the breath. Among others, the following is worthy of notice: A glass plate was covered with a few drops of twice distilled hydrochloric acid. I then breathed upon it about 65 times, always taking a full breath and expelling it with force on the glass-plate, which was afterwards held over a gaslight to evaporate the fluid and induce it to crystallize. We then subjected it to the microscope, and found the most beautiful crystals of hydrochlorate of ammonia, which resembled—though better marked—the crystals of hydrochlorate of ammonia which were placed on another plate in order to compare their appearance with those formed by the breath on muriatic acid.

The existence of ammonia in blood, is shown in this way: A receiver not entirely filled with blood, was covered by a plate wet with muriatic acid. The interspace being filled with the vapor of ammonia, forms then the crystals of ammonia. According to Dr. Richardson, the quantity of ammonia may be ascertained approximatively, by using choride of platinum, which will form with ammonia, yellow crystals, the weight denoting the quantity thus formed.

The existence of ammonia varies in the breath of different persons, according to the state of health, the same individual even, giving off sometimes more, s ometimes less. More is given off fasting than during digestion, and the air last expelled from the lungs during such expiration, contains the greatest proportion. I take with me an extract of his researches not yet published, in order to communicate it to the Congress of Physicians at Vienna.

Little of importance is going on, just now. Ferguson is grouse shooting in Sclotland, and Sir James is watching the movements of the Queen in Balmora ; Erichsen is rusticating at Scarboro, possibly trying experiments with the sponge on irrational animals, for he found out that he has to go down the throat, or suffer in the estimation of the lovers of truth. The train by which I go to Paris leaves directly, and interrupts further communication for the present. Yours, &c.

—*Am. Med. Monthly.* I. GLUCK.

